# Characteristic of Ultrastructure of Mice B16 Melanoma Cells under the Influence of Different Lighting Regimes

**DOI:** 10.3390/clockssleep4040056

**Published:** 2022-12-15

**Authors:** D. A. Areshidze, M. A. Kozlova, V. P. Chernikov, A. V. Borisov, D. V. Mischenko

**Affiliations:** 1 Avtsyn Research Institute of Human Morphology of Federal State Budgetary Scientific Institution “Petrovsky National Research Center of Surgery”, 117418 Moscow, Russia; 2 Institute of Problems of Chemical Physics, Russian Academy of Sciences (IPCP RAS), 142432 Chernogolovka, Russia

**Keywords:** melanoma, light pollution, circadian rhythms, melatonin, tumor growth, transmission electron microscopy

## Abstract

Circadian rhythms of physiological processes, constantly being in a state of dynamic equilibrium and plastically associated with changes in environmental conditions, are the basis of homeostasis of an organism of human and other mammals. Violation of circadian rhythms due to significant disturbances in parameters of main environmental effectors (desynchronosis) leads to the development of pathological conditions and a more severe course of preexisting pathologies. We conducted the study of the ultrastructure of cells of mice transplantable malignant melanoma B16 under the condition of normal (fixed) lighting regime and under the influence of constant lighting. Results of the study show that melanoma B16 under fixed light regime represents a characteristic picture of this tumor—predominantly intact tissue with safe junctions of large, functionally active cells with highly irregular nuclei, developed organelles and a relatively low content of melanin. The picture of the B16 melanoma tissue structure and the ultrastructure of its cells under the action of constant lighting stand in marked contrast to the group with fixed light: under these conditions the tumor exhibits accelerated growth, a significant number of cells in the state of apoptosis and necrosis, ultrastructural signs of degradation of the structure and functions, and signs of embryonization of cells with the background of adaptation to oxygen deficiency.

## 1. Introduction

Certain rhythmicity is characteristic for a significant number of processes occurring in mammalian organisms. The most important biological rhythms are circadian rhythms (CRs) with period of 24 ± 4 h.

The mammalian circadian system is hierarchically organized in a rather complex manner. The strict coordination of these rhythms with each other and with the rhythms of processes in the environment is the basis for maintaining homeostasis and ensuring the adaptive capabilities of an organism. The main factor which synchronizes an internal time with an external environment is light. Central oscillators (suprachiasmatic nuclei of hypothalamus, pineal gland), receiving external signals, regulate the operation of the peripheral “clocks”, which are present in all tissues and cells, but they, in turn, are a self-sustaining system in the absence of external signals [1,2]. 

The violation of the light–dark regime, so-called light pollution, is a significant factor in the disorganization of biorhythms. In the modern world, with its digitalization of all work processes and irregular working conditions due to social challenges, a person is inevitably exposed to abundant artificial lighting and is forced to shift the regime towards increasing the length of individual light day. This leads to the development of desynchronosis, disrupts the normal synchronization of endogenous rhythms with natural rhythms of light–darkness change, and prevents the synthesis of pineal melatonin. Light pollution causes shifts in most of the constants that characterize the homeostasis of organism and provokes accelerated aging and the development of a wide range of diseases, which is especially important in relation to an increase in the incidence of malignant neoplasms and a more severe course of tumor development processes [3].

At the molecular level, CRs are realized through several transcriptional–translational feedback loops, which include clock genes and corresponding proteins. The main clock genes are included in the *Bmal*, *Clock*, *Per*, *Cry* families, encode transcription factors, and form the main feedback loop [4,5]. There are several additional regulatory circuits in the cell that are necessary for the finer adjustment of CRs, which include RORa, REV-ERBa, and other transcription factors. Genes controlled by the “cell clock” regulate the processes of proliferation, metabolism, DNA replication and repair, and apoptosis [6,7]. A link between 10 hallmarks of cancer and clock genes is proven [8,9]. 

Since the period of circadian rhythms is 24 h, in order to maintain synchronization with the environment, the circadian rhythm should be daily entrained by Zeitgebers (external time-setting signals) [10]. In case of a serious mismatch of rhythms caused by a long-term and stable change in Zeigeber parameters, the state of desynchronosis occurs in an organism which leads to development of pathological conditions, a significant proportion of which are neoplasms, including melanoma, which is one of the most aggressive types of cancer [11].

Herewith, the relationship between CR disorders and the tumor process is not unilateral. While the disruption of circadian rhythms contributes to the development of tumors, the daily coordination of gene expression as such is, at the same time, basically disrupted in them [12,13]. In addition, the development of a tumor has a systemic negative effect on the organism, which causes a violation of the rhythmic parameters of its systems and organs among other things.

One of the factors that cause carcinogenesis is a decrease in the production of pineal melatonin due to the impact of excessive lighting. This phenomenon is also involved in the disruption of normal circadian rhythms, but in addition, melatonin has the described carcinoprotective effects and possesses oncostatic properties in various tumor models including melanomas [14]. 

Melanocytes are characterized by the presence of melanocyte-specific receptors, which include melanocortin-1 (MCR1) and melatonin receptors [15]. Pleiotropic bioactivities of melatonin are mediated through interactions with high-affinity membrane bound or nuclear receptors or through nonreceptor actions. Other important regulators of normal and malignant melanocyte activities which act through corresponding G-protein-coupled receptors are endothelines, histamine, corticotrophin-releasing hormone (CRH), catecholamines, and serotonin. It is described by a number of authors that loss of melatonin or its receptors may promote melanoma development and progression. Specific melatonin binding sites are found in mouse, hamster, and human melanoma lines [16,17].

A number of clinical studies had positive results with melatonin application in patients with metastatic malignant melanoma. Most recently, the tumorostatic effect of melatonin on human melanoma cell lines of different behavior was shown. The intensity of the tumorostatic response to melatonin is related to the cell-line-specific pattern of melanocyte cell surface and nuclear receptor expressions. It is known that effects of melatonin on melanogenesis inhibition, stimulation of DNA repair, and expression and activity of antioxidant enzymes (e.g., superoxide dismutase and catalase) are dependent on melatonin receptors phenotype [18,19,20]. An inhibitory effect of melatonin on melanogenesis was found in B16 melanoma [21]. Realization of the above-mentioned effects of the hormone is caused by its influence on homeostasis and circadian rhythms, inflammation, immunocyte cooperation, and cytokine production in a tumor microenvironment, gene expression and signaling pathways associated with angiogenesis, proliferation, and metastasis, metabolism, hypoxia and oxidative stress, apoptosis, and resistance to chemotherapy and radiation therapy. At the same time, mechanisms of antineoplastic effects of melatonin are still not fully investigated. Its effect on the activation of T-helper 1, an increase in the production of a number of cytokines (IL-2, IFN-γ, interleukin-6), decrease in VEGF receptor expression, activation of apoptosis in tumor cells, and reducing of telomerase activity are described [22].

As well as melatonin receptors being expressed in melanocytes in norm and in melanoma cells, there is the potential to mediate phenotypic actions on cellular proliferation and differentiation [23]. Moreover, receptor-independent activity of melatonin suggests that it may also play a protective role against UV-induced pathologies [24]. Both biosynthetic and biodegradative pathways for melatonin are described to be present in whole human skin and in melanoma cells [25].

In this regard, a large number of studies are devoted to the investigation of the effect of melatonin on tumor growth in violation of circadian rhythm in experimental models.

It has been established that CR disruption under conditions of excessive constant lighting promotes cancerogenesis, and an application of exogenous melatonin leads to the inhibition of tumor growth in cases of both intact and disrupted circadian rhythms [26,27,28,29,30]. However, a number of authors observe such an effect of melatonin only under standard illumination but note the stimulation of tumor growth by this hormone under constant illumination [31].

A number of studies described the relationship of constant lighting, i.e., deficiency of pineal melatonin, with the development of transplantable melanoma B16 [32,33]; the resistance of melanoma to chemotherapy under constant illumination conditions [34], as well as the antiproliferative and antimigratory effects of melatonin on melanoma cells are established [35,36].

At the same time, we did not find any studies on ultrastructural peculiarities of transplantable malignant melanoma cells under conditions of constant lighting. 

In this regard, we conducted a study of the ultrastructure of cells of transplantable malignant B16 melanoma under conditions of a fixed light regime and constant illumination.

## 2. Results

As a result of the study, we found that in animals kept under constant illumination, the body weight significantly increased, amounting to 24.50 ± 0.42 g against 22.16 ± 0.44 in the control, the same is observed for tumor mass (5.64 ± 0.28 g against 4.98 ± 0.27 g in control) and its volume—2.07 ± 0.37 cm^3^ in mice of control group and 5.70 ± 0.25 cm^3^ in the group kept under constant lighting.

### 2.1. Ultrastructure of Transplantable Malignant Melanoma B16 in Conditions of Fixed Light Regime

**Figure 1:**
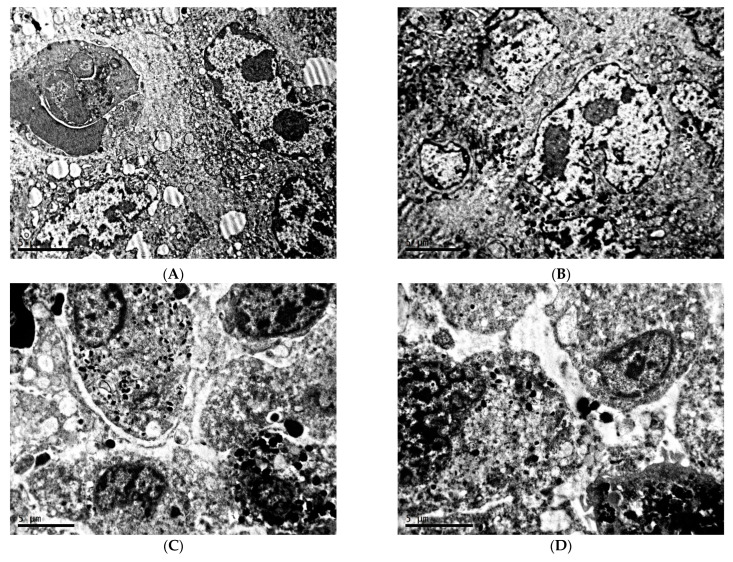
Morphological condition of tissue of transplantable malignant melanoma B16 under the influence of fixed light regime. TEM, (**A**–**D**)—×6700.

Melanoma B16 under fixed light regime represents predominantly intact tissue with safe cell junctions. Cells are 20–25 µm in diameter, polyhedral, irregular, or spindle-shaped. The highly irregular nuclei are characterized by an uneven contours of membrane with deep invaginations, electron-dense nucleoplasm, the presence of one or two nucleoli, and marginal condensation of small lumps of chromatin (Figure 1A,B). Melanin content in melanoma cells in this experimental group is relatively low (Figure 1C,D).

**Figure 2:**
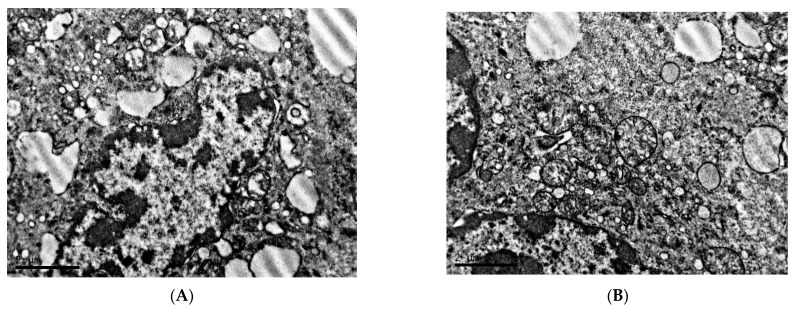
Organelles of cells of transplantable malignant melanoma B16 under the influence of fixed light regime. TEM, (**A**)—×10,000, (**B**)—×14,000.

The Golgi complex is well-developed in the cells; there is a moderate amount of mitochondria with a light matrix and small number of cristae. In the cytoplasm, there are a large number of vesicles and a significant number of ribosomes and lysosomes. In addition, the cytoplasm contains a large number of vacuoles, the contents of which—with uneven electron density, in most cases with a characteristic transverse striation—indicate that these vacuoles belong to the lipid fraction (Figure 2A,B).

**Figure 3:**
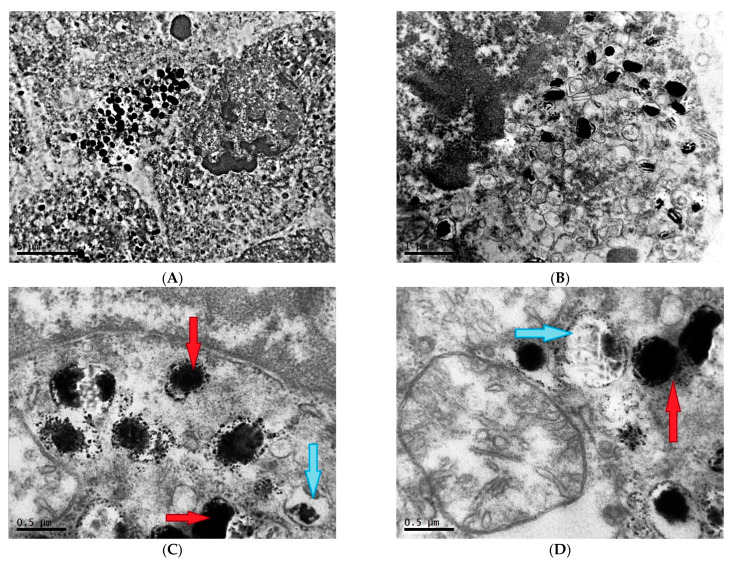
Melanosomes and mitochondria of cells of transplantable malignant melanoma B16 under the influence of fixed light regime. Blue arrows—premelanosomes, red arrows—mature melanosomes. TEM, (**A**)—×8000, (**B**)—×20,000, (**C**,**D**)—×40,000.

Spherical and elliptical melanosomes of various sizes are diffusely distributed in the cytoplasm (Figure 3A). Both mature melanosomes of uniform electron density, and the presence of oval-shaped premelanosomes with electron-dense material centrally located in the form of a strip are observed (Figure 3B). At high magnifications, the content of rather loose accumulations of melanin grains in the central region and along the periphery of the elementary membrane of mature melanosomes is noted (Figure 3C,D). 

**Figure 4:**
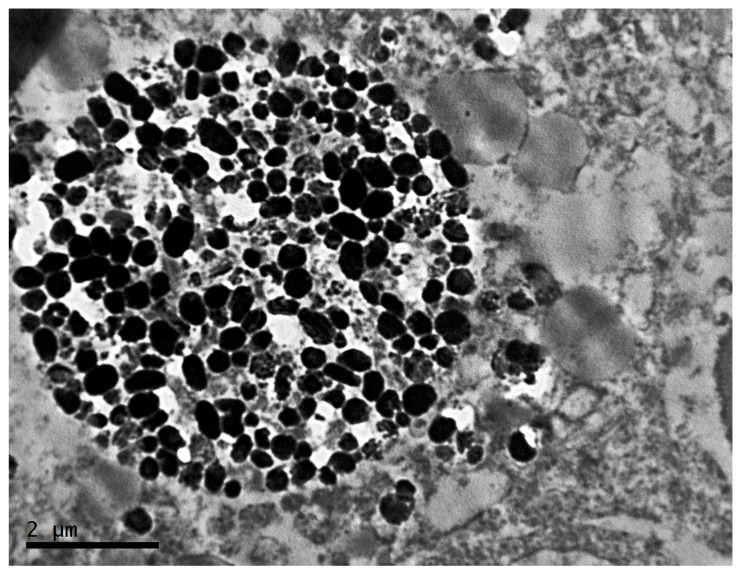
Spherical melanosome complex in cell of transplantable malignant melanoma B16 under the influence of fixed light regime. TEM, ×14,000.

These organelles are located mostly separately, but in single cases, the presence of spherical complexes containing melanosomes of different maturity is observed (Figure 4).

**Figure 5:**
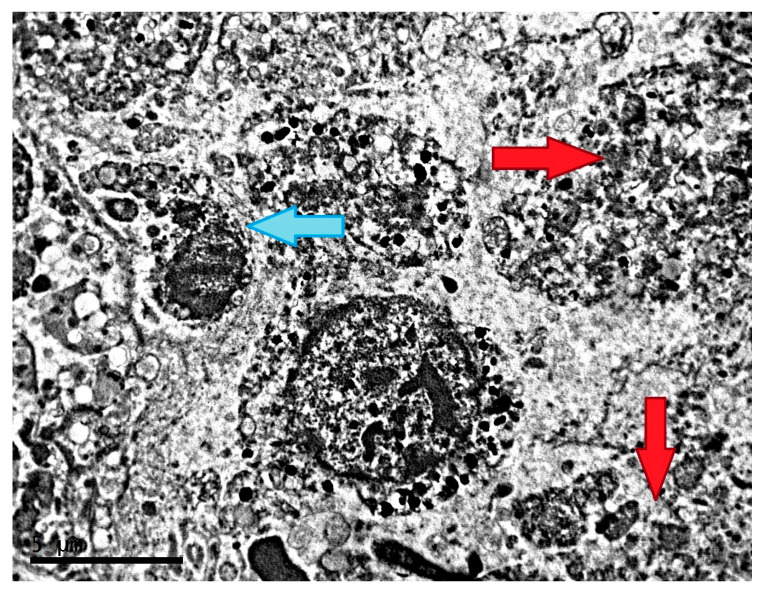
Cell death in transplantable malignant melanoma B16 under the influence of fixed light regime. Blue arrow—apoptosis (shrunken organelles and remnants of nuclear chromatin are visible), red arrows—necrosis. TEM, ×8000.

In some areas of the sample, both the presence of apoptotic bodies and cases of necrotic cell death are observed (Figure 5).

### 2.2. Ultrastructure of Transplantable Malignant Melanoma B16 in Conditions of Constant Lighting Regime

**Figure 6:**
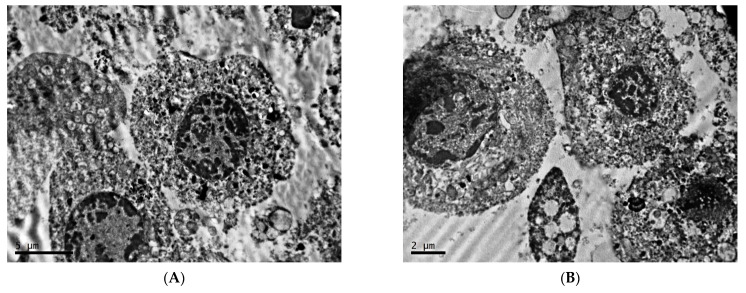
Morphological condition of tissue of transplantable malignant melanoma B16 under the influence of constant lighting. TEM, (**A**,**B**)—×6700.

In animals exposed to constant illumination, the tumor tissue pattern is represented by small (10–15 µm in diameter), round, non-connected cells; extreme looseness and edema of an extracellular matrix are noted (Figure 6A,B).

**Figure 7:**
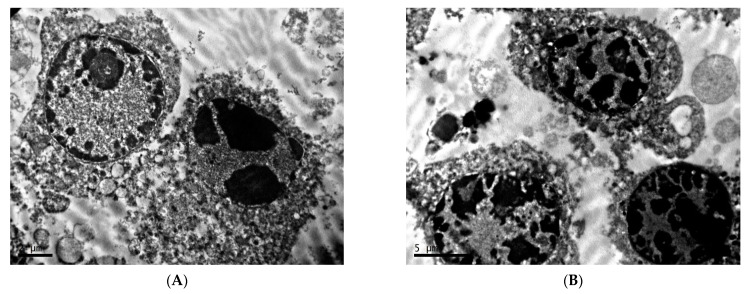

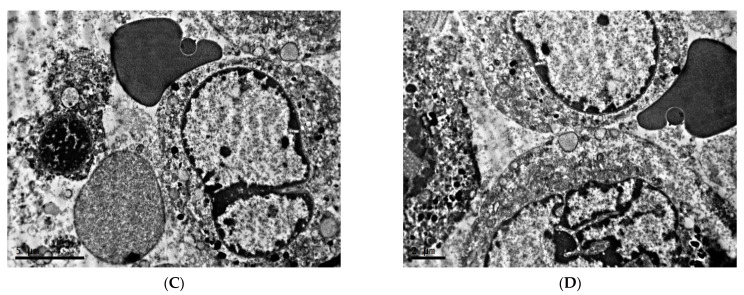
Characteristics of nuclei of cells of transplantable malignant melanoma B16 under the influence of constant lighting. TEM, (**A**,**D**)—×10,000, (**B**)—×6700, (**C**)—×8000.

Melanoma cells in this group are characterized by a significant increase in the nuclear–cytoplasmic ratio due to the pronounced decrease of the cytoplasm area; the number of organelles is reduced in comparison with the first group. In the nuclei of a significant number of cells, a fragmented distribution of condensed chromatin with rough lumpy structure is noted, which is characteristic of the beginning of the process of programmed cell death (Figure 7A,B). The presence of fragmented nuclei is also observed (Figure 7C,D).

**Figure 8:**
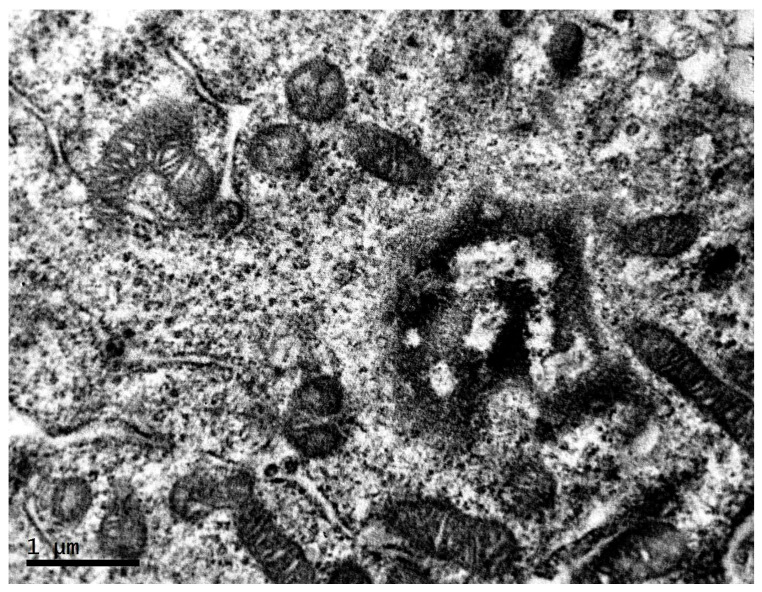
Organelles of cells of transplantable malignant melanoma B16 under the influence of constant lighting. TEM, ×20,000.

A significant number of ribosomes are observed. The endoplasmic reticulum is weakly expressed or vacuolated. The Golgi complex is poorly developed. In contrast to the cells of the first experimental group, in this group, there is practically no content of lipid vacuoles in the cells. The content of mitochondria in tumor cells in this group is higher than under fixed lighting. Mitochondria are smaller, with denser matrix and significantly greater number of cristae than was noted for the control group (Figure 8).

**Figure 9:**
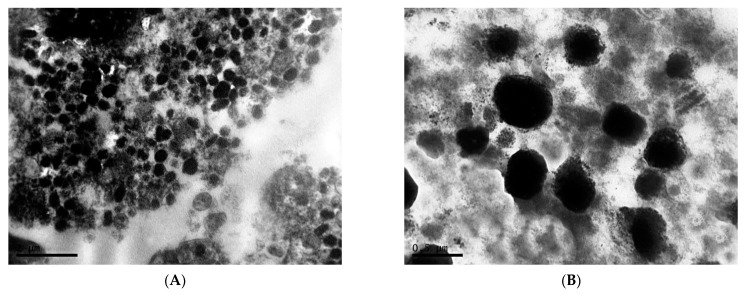
Characteristics of melanosomes of cells of transplantable malignant melanoma B16 under the influence of constant lighting. TEM, (**A**)—×14,000, (**B**)—×20,000.

The number of melanosomes in the cells of this group is significantly higher than in the control group, and proportion of mature melanosomes increases. In addition to an increase in the number, there is also a difference in the characteristics of the melanosomes themselves—in animals of this group, they are more dense and have a smooth surface (Figure 9A,B).

**Figure 10:**
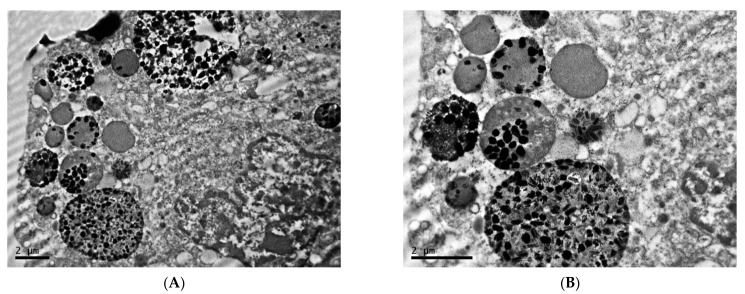

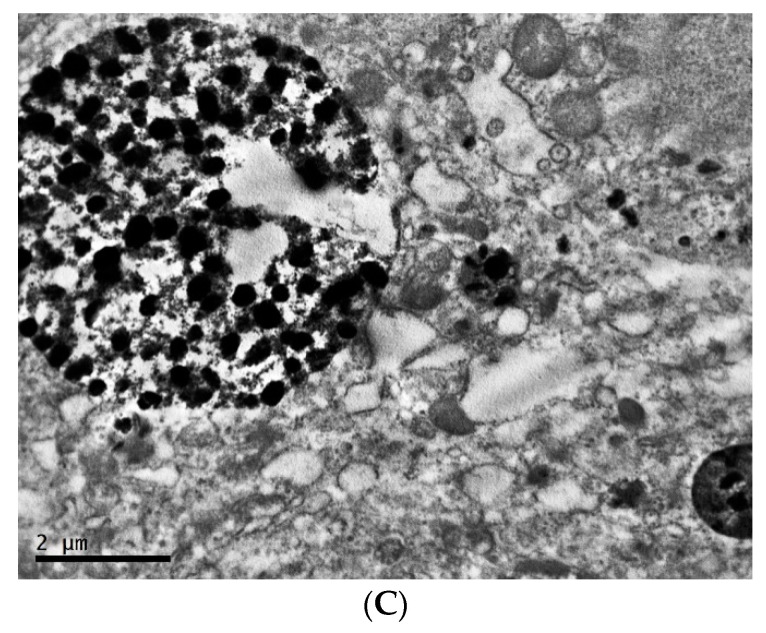
Melanosome complexes of cells of transplantable malignant melanoma B16 under the influence of constant lighting. TEM, (**A**)—×10,000, (**B**,**C**)—×14,000.

A characteristic feature of the cells of this group is the content of a large number of melanosome complexes in them. There is a presence of spherical formations of various sizes, surrounded by a membrane, which contain a large number of melanosomes of varying degrees of maturity (Figure 10A–C).

**Figure 11:**
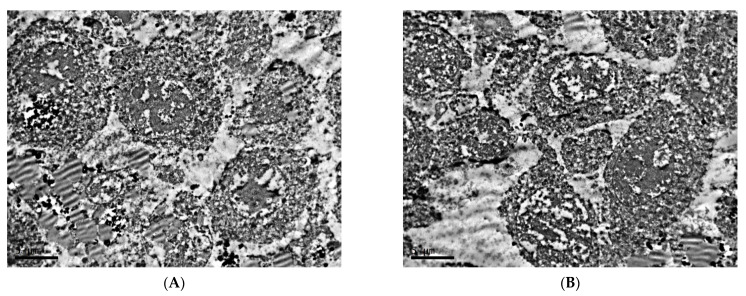
Groups of cells of transplantable malignant melanoma B16 in the state of necrosis under the influence of constant lighting. TEM, (**A**,**B**)—×5000.

Extensive areas of tumor tissue in this group represent bunches of cells in a state of necrosis—karyorrhexis progressing to karyolysis, lysis of organelles, and destruction of the plasmalemma are noted in groups of cells (Figure 11A,B).

## 3. Discussion and Conclusions

Being the interface between the organism and the external environment, the skin has complex defense mechanisms to counteract the deleterious effects of the physical, chemical, and biological stimuli, which affects not only immune and neuroendocrine mechanisms, pigmentary system, processes of DNA repair, or apoptosis of damaged cells, but also the molecular clock of the skin. The same is true for skin tumors, of which melanoma is the most complex, aggressive, and requiring detailed investigation [37]. The study of ultrastructural morphologic changes of cells of this tumor under the influence of socially significant chronodisruptors is necessary for appreciation of the carcinogenic process and development of therapeutic strategies in melanoma treatment.

The conducted study allowed us to establish the increase of weight and volume of tumor, the increase of body weight caused by it, and a number of differences in the ultrastructure of melanoma cells in animals kept under constant illumination from those in mice that were kept under a fixed light regime.

We established that in conditions of melatonin deficiency due to constant illumination, both ultrastructural signs characteristic for melanoma itself and specific ones caused precisely by the lack of melatonin are observed.

The presence of marked irregularity of nuclei, highly developed endoplasmic reticulum and multiple, dilated Golgi complex, and varying amounts of dense melanin granules in melanosomes of varying size are characteristic for melanomas. Although the number of mitochondria varies in individual neoplasms, malignant cells generally have diminished oxidative phosphorylation and mitochondria with short stubby cristae. Malignant melanoma cells are often large, polyhedral, and black, but cells show much variability in shape and pigmentation. Some melanomas are composed of elongate, stellate-shaped cells. Some contain only blastic cells, which produce little pigment [38]. 

We noted that in tumors of animals kept in constant light, the cells are small and rounded, which is due to the fact that they exhibit high mitotic activity without reaching the synthetic phase of the cell cycle, i.e., there is a well-known phenomenon of morphological embryonization (dedifferentiation, anaplasia) of cells in neoplasms [39,40]. High nuclear–cytoplasmic ratio, small size (1–2 erythrocyte sizes), round shape, and low differentiation are characteristic of actively growing highly aggressive malignant tumors [41]. There are many described hallmarks of malignant cancer cells, such as significant loss of polarity and organization, elevated nuclear cytoplasmic ratio and nuclear volume density, expression of tumor-specific antigens, gain of tissue-specific functions such as protein secretion at physiologic levels, and resistance to chemotherapy drugs [42]. Thus, the embryonicization of B16 melanoma cells in animals exposed to constant light indicates their accelerated development (tumor growth) up to metastatic stages [43].

The observed increase in the number of small mitochondria is explained by the fact that although in general there is a decreased dependence of the neoplastic cell on mitochondrial respiration (which confers a selective advantage to begin growth in the tumor mass—a poorly vascularized, oxygen-deficient environment), metabolic adaptation calls on mitochondrial function and draws on the mitochondrial reserve to meet increasing needs [44]. A number of studies showed an increase in “spare respiratory capacity” of melanoma cells—the mitochondrial capacity to meet extra energy requirements, beyond the basal level, which occurred in response to acute cellular stress or heavy workload [45]. In particular, this occurs due to activation of PGC1α-dependent mitochondrial biogenesis, regulation of activity of enzymes involved in the respiratory chain by Sirtuin-3 directly and through an increased activity of the antioxidant enzyme SOD2, required for maintenance of spare respiratory capacity [46,47].

The increase in melanin content in tumor cells in the second group is explained by the fact that not only ultraviolet radiation, but also visible light can lead to the development of hyperpigmentation. Visible light stimulates tyrosinase and dopachrome tautomerase to form a protein complex that induces sustained tyrosinase activity, which results in clinically identifiable pigmentation [48]. Justifying the increase in the content of melanin in the cells of the animals of the experimental group, we should also mention that melanin exhibits antioxidant effects by scavenging free radicals, while the absence of melatonin due to dark deprivation leads to the development of pronounced oxidative stress, including in the skin [49,50,51]. 

The proliferating melanoma cells show an accumulation of melanosomes, even though only a few cells expressed melanin—this means the initial stage of formation of these organelles, their inner structure, which precedes the formation of melanin. It is suggested that most of the melanosomes in the proliferative phase are amelanotic, i.e., in stage I and II [52]. Starting in stage III, in stage IV melanosomes, melanin deposition is completed and it occludes the internal structure of the melanosome [53]. In our experiment, there are more immature melanosomes in the control group, and the melanin content in mature melanosomes is lower, so they are looser and more transparent. The melanomas in this group are probably mostly stage I and II. In the group of animals exposed to constant light, mature melanosomes prevail in number and contain more melanin in them. According to a significant number of studies, melanization is increased in relation to oxidative stress and associated with it in bilateral way, so the increase of melanin content and more high maturity of melanodome is a reflection of more pronounced oxidative stress and DNA damage caused by it (due to lack of pineal melatonin production), than in the control group [54,55,56]. 

The presence of melanophages and vesicular structures associated with aberrant melanosomes has long been described in primary and metastatic lesions [57]. Complex melanosomes, which are membrane-bound lysosomes filled with degenerative primary melanosomes, arise as a result of autophagy [58]. 

Studies using cytochemical indicators have shown that melanosomes can fuse with each other or with lysosomes to form melanosome complexes. Melanosome complexes are found not only in Harding–Passi melanoma cells, but also in mice B16 melanoma. The formation of melanosome complexes is part of a wide range of subcellular changes during intracytoplasmic autodegradation of melanoma cells [59].

Electron microscopy revealed phagocytic structures in melanocytic lesions quite a long time ago [60]. In the early 1980s, it was already discovered that nevus, lentigo simplex, and skin melanomas contain large melanosome complexes incorporated into vesicular structures and reflecting various stages of degradation [61]. These melanized autophagosomes are now thought to be responsible for the so-called coarse melanin, which is the cause of highly hyperpigmented areas in melanoma samples [62]. The molecular basis of formation and, more importantly, the role of these melanin-containing autophagosomes are not fully understood for date. It is interesting that hyperpigmented melanomas are characterized by marked infiltration by melanophages that share ultrastructural and immunohistochemical features with melanoma cells. Based on these similarities and the observation that melanoma cells can exhibit a pronounced phagocytic phenotype [63], a model according to which the fusion of melanoma and macrophages can contribute to the progression of melanoma was proposed [64].

The study of changes in tumors under the action of chronodisruptors is an extremely topical subject in the modern world and requires the closest attention. The aim of our study was to establish the fine ultrastructural features of melanoma cells in standard conditions and in conditions of absence of endogenous pineal melatonin due to constant lighting.

As mentioned above, it has been shown by a number of studies that melatonin treatment has significant influence on metabolic and proliferation activity of melanocytes in norm and pathology. Our study, being a natural continuation of previous physiological and biochemical studies of other researchers, indicates that B16 melanoma exhibits accelerated growth in constant light conditions, accompanied by significant ultrastructural changes. On the one hand, a significant number of cells in the state of apoptosis and necrosis, as well as cells with ultrastructural signs of degradation of the structure and functions, are observed in the tumor. On the other hand, the ultrastructure of melanocytes in tumors of animals kept under constant lighting indicates accelerated tumor growth due to more pronounced embryonicization of melanoma cells, as well as signs of adaptation to oxygen deficiency.

## 4. Materials and Methods

### 4.1. Object of Study 

The study was conducted on male BDF1 hybrid mice (*n* = 50) at the age of 8 weeks, with body weight of 21–22 g, taken from USF «Vivarium and Animal Housing Group of Screening and Preclinical Studies Unit of Federal Research Center of Problem of Chemical Physics and Medicinal Chemistry RAS». The animals were kept in standard laboratory conditions, at a temperature of 20–22 °C and a relative humidity of 60–70%, had free access to drinking water and briquetted food. Keeping of animals and experiments were performed in accordance with the European Convention for the Protection of Vertebrate Animals used for Experimental and other Scientific Purposes (Strasbourg, 18 March 1986). This research was approved by the Bioethical Committee of the Federal State Budgetary Scientific Institution “Research Institute of Human Morphology”, protocol No. 34 (10) (14 March 2022).

### 4.2. Design of Study

Mice were divided into 2 equal groups.

The control group (*n* = 25) (control) was kept under fixed light regime (light:dark/10:14 h with lights on at 8:00 and off at 18:00).

The experimental group (*n* = 25) was kept under the regime of constant lighting.

Animals were kept under artificial lighting with fluorescent lamps. The luminous flux per cage area unit was 150 lux, in accordance with the Russian sanitary standards for working premises lighting [65].

B16/F10 melanoma was transplanted to each animal by standard subcutaneous injection of 0.5 mL of a suspension of tumor tissue in medium 199 at a dilution of 1:10 by weight, with a 1.2 × 40 needle, into the area of the left flank closer to the back, after anesthesia by diethyl ether [66,67].

Withdrawal of animals from the experiment was carried out on the 15th day after tumor transplantation. Euthanasia was carried out by the method of cervical dislocation. After euthanasia, the tumor was taken.

The mass of animals and tumors was determined. Tumor volume was determined by the formula: V = π/6 × D1 × D2 × D3, where D1—length, D2—width, and D3—height of the tumor in in centimeters.

### 4.3. Electron Microscopy

Melanoma samples of 2 mm^3^ size were fixed with a 2.5% solution of glutaraldehyde in 0.1 M phosphate buffer (pH 7.4), additionally fixed in a 1% solution of osmium tetroxide (OsO_4_), dehydrated in ethanol according to the generally accepted scheme, contrasted with 1% uranyl acetate in 70% ethanol during dehydration and poured into the epon–araldite mixture according to the standard procedure.

Ultrathin sections were obtained on an LKB-III ultramicrotome (LKB Produkter, Sweden), the sections were additionally counterstained with lead citrate according to the Reynolds method and viewed with a JEM-100CX transmission electron microscope (JEOL, Tokyo, Japan). The photofixation of preparations was carried out using a Gatan ES500W Erlangshen camera (Model 782), (Gatan Inc., Pleasanton, CA, USA) at magnifications of ×5000, ×6700, ×8000, ×10,000, ×14,000, ×20,000 and ×40,000.

The shapes of the nuclei of B16 melanoma cells and condition of their organelles were evaluated, and the presence of lipid vacuoles was revealed during transmission electron microscopy.

### 4.4. Statistical Evaluation

The obtained data were analyzed using the GraphPad Prism 6.0 program by calculating average values, standard deviation, and arithmetic mean error. The data are presented as mean *±* SEM. To assess the significance of differences, the Student’s *t*-test was used. Changes were considered reliably significant at *p* ≤ 0.05.

## Data Availability

The data presented in this study are available within the article text, tables, and figures.
